# 儿童异基因造血干细胞移植后人类细小病毒B19感染的血液学表现

**DOI:** 10.3760/cma.j.issn.0253-2727.2021.08.007

**Published:** 2021-08

**Authors:** 陆阳 张, 芳 刘, 霞 陈, 小燕 张, 媛媛 任, 然然 张, 文钰 杨, 晔 郭

**Affiliations:** 中国医学科学院北京协和医学院血液病医院（中国医学科学院血液学研究所）儿童血液病诊疗中心，实验血液学国家重点实验室，国家血液系统疾病临床医学研究中心，天津 300020 State Key Laboratory of Experimental Hematology; National Clinical Research Center for Blood Diseases; Children's Blood Disease Diagnosis and Treatment Center, Institute of Hematology & Blood Disease Hospital, Chinese Academy of Medical Sciences & Peking Union Medical College, Tianjin 300020, China

**Keywords:** 人类细小病毒B19, 造血干细胞移植, 儿童, Human parvovirus B19, Hematopoietic stem cell transplantation, Children

## Abstract

**目的:**

探讨异基因造血干细胞移植（allo-HSCT）患儿造血重建后人类细小病毒B19（HPV-B19）感染的血液学表现。

**方法:**

对9例allo-HSCT后合并HPV-B19感染的患儿进行回顾性分析。

**结果:**

9例患儿占同期接受allo-HSCT患儿的8.04％（9/112），男8例，女1例，中位年龄9（3～13）岁，均采取清髓性预处理方案。HPV-B19感染中位时间为移植后61（36～114）d。allo-HSCT并发HPV-B19感染患儿血液学表现具有异质性，9例患儿以血红蛋白伴网织红细胞下降为主要特点，7 d内网织红细胞比例、绝对值下降幅度中位数分别为90.4％（24.7％～98.7％）、90.7％（18.6％～99.0％）。除常见红系造血停滞表现外，allo-HSCT后合并HPV-B19感染的患儿还具有非红系的血象及骨髓变化：5例患儿外周血出现中性粒细胞下降，但骨髓涂片未见粒系增生受抑；6例患儿骨髓涂片查见巨核系增生减低，其中5例患儿外周血血小板下降。同时，allo-HSCT造血重建后合并HPV-B19感染的患儿骨髓红系受抑并非必要表现，9例患儿虽然均出现血红蛋白下降，但仅5例患儿骨髓红系增生减低。

**结论:**

血液病患儿allo-HSCT造血重建后合并HPV-B19感染的血液学表现具有异质性，血红蛋白伴网织红细胞下降对HPV-B19感染早期诊断可能具有重要意义。

## Abstract (en)

**Objective:**

To retrospectively analyze hemogram and bone marrow characteristics of pediatric patients infected with parvovirus B19（HPV-B19）after hematopoietic reconstitution following allo-hematopoietic stem cell transplantation.

**Methods:**

The clinical course of nine patients with HPV-B19 infection, including hemogram and bone marrow smear analysis during infection, were retrospectively analyzed.

**Results:**

Despite the hematological heterogeneity, all patients exhibited reduced hemoglobin levels accompanied by reticulocytes. The proportion and absolute count of reticulocytes decreased by 90.4％（24.7％–98.7％）and 90.7％（18.6％–99.0％）, respectively, in one week. Additionally, five patients manifested a decline in neutrophil granulocyte count in peripheral blood whereas granulocytic hypoplasia was not observed in bone marrow. Furthermore, six patients exhibited megakaryocytic hypoplasia in bone marrow, including five patients with decreased platelet counts in peripheral blood. Importantly, only some patients exhibited erythroid hypoplasia although all patients exhibited a decline in hemoglobin in peripheral blood. Erythroid hypoplasia in bone marrow was present in five patients.

**Conclusion:**

There was heterogeneity in hemogram and bone marrow smear characteristics among pediatric patients infected with HPV-B19 following allo-hematopoietic stem cell transplantation. Anemia accompanied by decreased reticulocyte count should prompt screening for HPV-B19 in these patients

人类细小病毒B19（HPV-B19）系细小病毒科红病毒属，是一种人群感染率较高的单链DNA病毒，其血清学阳性率在学龄前儿童、年轻成人、老年人中分别为15％、50％、85％[1]。HPV-B19感染在造血干细胞移植（HSCT）患儿中也有较高的发生率[2]–[3]。本研究回顾性分析9例allo-HSCT造血重建后合并HPV-B19感染血液病患儿的临床特点、治疗反应及转归，旨在加深对血液病患儿allo-HSCT后HPV-B19感染的认识，为临床诊断和鉴别诊断提供借鉴。

## 病例与方法

一、病例

本研究纳入2018年1月1日至2020年11月30日在中国医学科学院血液病医院儿童血液病诊疗中心行allo-HSCT且造血重建后合并HPV-B19感染的儿童血液病患者。纳入标准：allo-HSCT造血重建后发生不明显原因血细胞下降，外周血HPV-B19-DNA检测阳性[4]。本研究经中国医学科学院血液病医院伦理委员会审查通过（批件号：HG2021003-EC-1）。

二、HPV-B19检测方法和治疗方案

采用RT-PCR的方法检测患儿外周血HPV-B19-DNA，检测范围（1×10^3^～1×10^8^）拷贝/ml。在首次检测阳性后，给予静脉免疫球蛋白（IVIg）治疗，同时联合抗病毒治疗以及免疫抑制剂减量。

三、HPV-B19感染后外周血三系受累标准

红系受累：血红蛋白降幅≥20 g/L或非输血依赖的患儿出现输血依赖，并排除失血、溶血、营养性贫血等原因。血小板受累标准：确诊HPV-B19感染，在红系下降基础上血小板计数下降>50％或出现血小板输注依赖。粒系受累：确诊HPV-B19感染，在红系下降基础上发生粒细胞减少或粒细胞缺乏。血小板及粒系受累同时排除其他因素。

四、随访

以HPV-B19-DNA首次阳性为随访起始时间，随访截止时间为2020年11月30日，中位随访时间为384（159～521）d。随访资料来自门诊/住院病历和电话随访记录。

## 结果

一、一般资料

9例患儿占同期接受allo-HSCT患儿的8.04％（9/112），男8例，女1例，中位年龄9（3～13）岁，均采取清髓性预处理方案。HPV-B19感染中位时间为移植后61（36～114）d。7例患儿首次HPV-B19-DNA定量检测>1×10^8^拷贝/ml（表1）。

例1获得中性粒细胞、血小板植入后未脱离红细胞输注依赖［网织（0.002～0.007）×10^12^/L红细胞0.05％～0.27％，绝对计数］，其余8例患儿均获得完全造血重建。HPV-B19感染前中位网织红细胞比例为4.1％（3.50％～8.90％），中位网织红细胞绝对计数为0.118（0.096～0.274）×10^12^/L，均明显高于正常水平。详见表1。

**表1:** 人类细小病毒B19（HPV-B19）感染患儿一般资料及移植特征

例号	年龄（岁）	性别	诊断	移植类型	GVHD预防方案	急性GVHD	移植后HPV-B19-DNA初次阳性时间（d）	初次HPV-B19-DNA定量（拷贝/ml）	基础Ret（％）	基础Ret（×10^12^/L）
1	11	女	SAA	haplo-HSCT	CsA+MMF+MTX	Ⅰ	36	>1×10^8^	NA	NA
2	13	男	AML	haplo-HSCT	CsA+MMF+MTX	无	114	>1×10^8^	4.07	0.100
3	6	男	AML	UCBT	CsA+MTX	Ⅱ	103	>1×10^8^	3.50	0.096
4	7	男	NSAA	haplo-HSCT	CsA+MMF+MTX	无	96	3.8×10^5^	7.63	0.235
5	10	男	MDS	UCBT	CsA+MMF+MTX	Ⅳ	67	>1×10^8^	4.61	0.132
6	5	男	AML	UCBT	CsA+MMF	Ⅳ	59	>1×10^8^	3.96	0.115
7	13	男	ALL	UCBT	CsA+MMF+MTX	Ⅳ	61	>1×10^8^	4.15	0.121
8	3	男	JMML	haplo-HSCT联合UCBT	PT-Cy+FK506+MMF	Ⅳ	47	>1×10^8^	4.11	0.102
9	9	男	MDS	haplo-HSCT	CsA+MTX	Ⅳ	48	2.33×10^3^	8.90	0.274

注：SAA：重型再生障碍性贫血；NSAA：非重型再生障碍性贫血；AML：急性髓系白血病；ALL：急性淋巴细胞白血病；MDS：骨髓增生异常综合征；JMML：幼年型粒单核细胞白血病；haplo-HSCT：单倍型造血干细胞移植；UCBT：脐血干细胞移植；CsA：环孢素A；MMF：霉酚酸酯；MTX：甲氨蝶呤。Ret：网织红细胞，参考值0.50％～1.50％、（0.024～0.084）×10^12^/L

二、allo-HSCT后HPV-B19感染患儿血液学表现

1. HPV-B19感染后网织红细胞及血红蛋白变化：9例患儿以贫血伴网织红细胞下降为主要特点，HPV-B19感染后患儿网织红细胞下降幅度、速度明显大于血红蛋白。例1移植后网织红细胞持续偏低，其余8例患儿均出现网织红细胞比例和绝对计数快速、明显下降，网织红细胞开始下降后7 d内网织红细胞比例、绝对计数降幅中位数分别为90.4％（24.7％～98.7％）、90.7％（18.6％～99.0％）。相应时间段内，患儿血红蛋白下降不明显或尚未开始出现下降（表2）。8例可评价患儿中6例血红蛋白与网织红细胞同步下降，2例血红蛋白下降迟于网织红细胞下降（1例延迟47 d，另1例延迟7 d）。

2. HPV-B19感染后血象及骨髓细胞形态学变化：除常见红系造血受抑表现外，此类患儿还具有非红系受抑的血液学改变。9例患儿中3例外周血出现红系、粒系及血小板三系下降，2例出现红系、粒系下降，2例表现为单纯红系下降，2例出现红系、血小板下降（表2）。

**表2:** 9例allo-HSCT后HPV-B19感染患儿血象及骨髓细胞形态学结果

例号	HGB最低值（g/L）	7d内Ret比例降幅（％）	7d内Ret绝对计数降幅（％）	外周血受累系别	HPV-B19感染时骨髓象
1	持续红系输注依赖	NA	NA	红系、血小板	E＝0.5％，G＝90.5％，全片巨核细胞3个
2	54	87.6	87.2	三系	G＝65％，红系、巨核系缺如
3	58	24.7	18.6	三系	E＝8％，G＝44％，全片巨核细胞4个
4	75	45.7	42.5	红系、粒系	E＝1％、可见巨大原始红细胞，G＝70％，全片巨核细胞12个
5	53	96.1	97.3	三系	E＝33％，G＝57.5％，巨核系缺如
6	58	93.2	94.2	红系	E＝1.5％，G＝58.5％，全片巨核细胞11个
7	69	94.3	95.4	红系	E＝23.5％，G＝54.5％，全片巨核细胞1个
8	57	98.7	99.0	红系、粒系	E＝17.5％，G＝72.5％，全片巨核细胞60个
9	44	49.8	61.7	红系、血小板	E＝51.5％，G＝42％，巨核系缺如

注：Ret：网织红细胞；E：红系比例，G：粒系比例。正常骨髓涂片参考值：红系占有核细胞15％～25％，粒系占有核细胞45％～70％，全片巨核细胞7～136个；NA：不适用

而骨髓细胞形态学改变具有多样性。例1红系、巨核系受抑，与外周血表现一致，其余8例患儿外周血改变与骨髓不完全一致。5例患儿外周血中性粒细胞下降，但骨髓涂片均未见粒系增生受抑；6例患儿骨髓巨核系增生减低，其中5例出现外周血血小板减少。全部9例患儿均出现贫血，但仅5例患儿骨髓红系增生减低，其余4例患儿中红系增生正常、偏高各2例。仅1例患儿骨髓涂片中见到巨大原始红细胞。详见表2。

3. HPV-B19感染治疗后血象及骨髓细胞形态学改变：9例患儿IVIg中位剂量为0.30（0.09～0.40）g·kg^−1^·d^−1^，中位疗程为18（9～35）d。在IVIg治疗后9例患儿均在短期内获得网织红细胞及血红蛋白恢复。以开始IVIg治疗为起点，7例患儿网织红细胞比例恢复至≥1％、绝对值恢复至≥0.024×10^12^/L的中位时间均为7（6～16）d，8例患儿网织红细胞比例、绝对值恢复到HPV-B19感染前水平的中位时间分别为17（6～35）d、16.5（6～35）d。9例患儿血红蛋白升高≥10 g/L中位时间为6（5～28）d，≥20 g/L中位时间为14（6～133）d。

8例患儿中，7例骨髓红系比例由治疗前的8.0（0～51.5％）增至治疗后的42.0％（12.5％～55.0％）、巨核细胞数由治疗前的3（0～60）个/全片增加至20（3～101）个/全片，2例患儿发生HPV-B19感染复发，再次IVIg治疗仍有效。详见表3。

**表3:** 9例异基因造血干细胞移植后人类细小病毒B19感染患儿静脉免疫球蛋白（IVIg）用量、疗效及转归

例号	IVIg用量	Ret ≥1％（d）	Ret ≥0.024×10^12^/L（d）	Ret比例恢复到基础水平（d）	Ret绝对计数恢复到基础水平（d）	HGB≥10g/L（d）	HGB≥20g/L（d）	转归
1	0.3g·kg^−1^·d^−1^×18d	6	6	NA^c^	NA^c^	5	7	有效
2	0.09g·kg^−1^·d^−1^×17d	11	11	18	32	5	21	有效
3	0.3g·kg^−1^·d^−1^×35d	NA^a^	NA^b^	16	14	5	14	复发
4	0.18g·kg^−1^·d^−1^×17d	11	11	18	32	28	133	有效
5	0.3g·kg^−1^·d^−1^×19d	7	7	8	8	6	6	复发
6	0.4g·kg^−1^·d^−1^×9d	16	16	19	19	5	11	有效
7	0.27g·kg^−1^·d^−1^×24d	6	6	8	8	6	38	有效
8	0.4g·kg^−1^·d^−1^×16d	6	6	6	6	7	9	有效
9	0.2g·kg^−1^·d^−1^×28d	NA^a^	NA^b^	35	35	9	32	有效

注：Ret：网织红细胞；NA：不适用；^a^细小病毒B19感染期间最低值仍≥1%；^b^细小病毒B19感染期间最低值≥0.024×10^12^/L；^c^移植后持续红系输注依赖、Ret比例及绝对值持续偏低

## 讨论

1975年，Cossart等[5]首次报告HPV-B19。20世纪80年代，先后有学者描述了健康志愿者HPV-B19感染的临床、血象及骨髓象变化[6]–[7]。Young等[8]在同一时期证明了HPV-B19对红系造血前体细胞的细胞毒作用。因此，多数文献描述HPV-B19急性感染的典型骨髓表现为红系增生减低、红系前体细胞缺如、偶见巨大红系原始细胞[9]–[10]。器官移植后HPV-B19感染的最早报道见于1986年肾移植患者[11]。目前，HSCT患者合并HPV-B19感染以个案报道较多[12]–[16]。既往文献报道，单倍型移植患者外周血HPV-B19-DNA阳性率高于同胞全相合移植患者（16.1％对4.2％，*P*＝0.04）[17]。也有文献就不同来源移植物HPV-B19-DNA进行分析，发现骨髓阳性率（30例中4例）高于无关脐带血（34例中3例）[18]，但是并未进一步评价移植患者是否存在HPV-B19感染相关症状。allo-HSCT后HPV-B19感染的高危因素可能与其他病毒感染类似（与移植前后免疫状态有关），但相较于人疱疹病毒6型（HHV6）、巨细胞病毒（CMV）、EB病毒（EBV）等移植后较常见的病毒感染[19]–[20]，目前尚缺乏其感染高危因素的进一步报道。

成人HSCT患者合并HPV-B19感染多发生于移植后3个月或以后[13],[21]。本组血液病患儿移植后HPV-B19感染的中位诊断时间为移植后61（36～114）d，可能与成人HPV-B19血清学阳性率较高、血清抗体在移植后早期仍可提供保护有关。

以往研究显示，在免疫功能正常和免疫功能缺陷人群，HPV-B19感染均可引起外周血三系细胞减少[1],[4],[6],[13],[22]，本组移植后血液病患儿的HPV-B19感染与以上报道一致。但本组患儿血红蛋白伴网织红细胞下降为其共同特点，且网织红细胞下降较血红蛋白下降更快、更明显。7 d内网织红细胞比例、绝对值下降幅度中位数分别为90.4％（24.7％～98.7％）、90.7％（18.6％～99％）；8例患儿中，6例血红蛋白与网织红细胞同步下降，2例血红蛋白下降迟于网织红细胞下降，提示移植造血重建后出现血红蛋白伴网织红细胞下降可能有助于早期确诊HPV-B19感染。

allo-HSCT造血重建后HPV-B19感染患儿的骨髓同样具有非红系受抑的表现。本研究中，6例患儿骨髓巨核系增生减低，提示HPV-B19感染除累及红系外，巨核系同样易受累及。既往报道中，接受自体造血干细胞移植（auto-HSCT）的多发性骨髓瘤患者HPV-B19感染以血小板减少为主要表现[22]。巨核系增生受抑制可能与血小板表面P抗原的存在、HPV-B19非结构蛋白对巨核细胞的细胞毒作用有关[22]–[24]。既往报道提示HPV-B19可引起骨髓三系增生受抑[25]–[27]，但本研究中骨髓改变以红系、巨核系造血受抑为主，即使外周血中性粒细胞减少者，骨髓中也未观察到髓系造血受抑，可能与粒系前体细胞对病毒感染有较低敏感性相关[7]。

本研究中，5例患儿骨髓涂片见红系增生减低，但2例患儿红系增生正常，还有2例患儿红系增生偏高。Crook等[9]曾报道8例免疫功能缺陷（获得性免疫缺陷综合征、白血病化疗、地中海贫血脾切除术后）患者HPV-B19感染后骨髓红系增生可不降低、且核内包涵体常见，作者认为骨髓无红系前体细胞缺如、且红系各个阶段可见核内包涵体，原因在于这些患者免疫功能缺陷。机体缺陷的免疫功能使病毒复制被阻断、但并不能清除病毒，红系前体细胞并没有因HPV-B19感染而凋亡，且继续发育，因此骨髓红系可无受抑表现，但外周血表现为网织红细胞下降[9]。本研究同样观察到患儿骨髓红系增生可不受抑制、但网织红细胞下降，不同的是在普通光学显微镜下，并未在患儿骨髓涂片中见到核内包涵体，其发生原因是否相同值得继续研究。移植后患儿HPV-B19感染多样性的血象、骨髓细胞形态变化特点，为HSCT后血细胞减少患者的诊断和鉴别诊断提供了新的思路。

治疗后网织红细胞比例及绝对值恢复到正常水平的中位时间为7 d，这与既往免疫缺陷患者治疗反应的报道一致[15],[28]。本研究结果显示，即使HPV-B19感染期间红系、巨核系增生正常的患儿，治疗有效者仍可观察到红系及巨核系增生进一步改善。

综上，本组病例结果显示，儿童血液病患者allo-HSCT造血重建后合并HPV-B19感染的血液学表现具有一定异质性且以贫血伴网织红细胞下降为主要特点，快速、明显的网织红细胞下降对HPV-B19感染的筛查可能具有重要意义。
